# Microarray analysis of long noncoding RNA and mRNA expression profiles in human macrophages infected with *Mycobacterium tuberculosis*

**DOI:** 10.1038/srep38963

**Published:** 2016-12-14

**Authors:** Xiaofan Yang, Jiahui Yang, Jinli Wang, Qian Wen, Hui Wang, Jianchun He, Shengfeng Hu, Wenting He, Xialin Du, Sudong Liu, Li Ma

**Affiliations:** 1 Institute of Molecular Immunology, School of Laboratory Medicine and Biotechnology, Southern Medical University, Guangzhou 510515, China; 2 Guangdong Provincial Key Laboratory of Tropical Disease Research, School of Public Health, Southern Medical University, Guangzhou 510515, China

## Abstract

Macrophages play a crucial role in the control and elimination of invading *Mycobacterium tuberculosis* (Mtb), and also serve as the major residence for Mtb. However, the interaction between macrophages and Mtb remains to be clearly determined. Although long noncoding RNAs (lncRNAs) have emerged as key regulators in many biological processes, their roles in anti-mycobacterial responses of macrophages remain to be elucidated. Here, we applied microarray analysis to examine lncRNA and mRNA expression profiles in human primary macrophages after 72 h of infection with H37Ra or H37Rv. Our results revealed that many lncRNAs were differentially expressed in macrophages after H37Ra or H37Rv infection, indicating a possible role for lncRNAs in immune responses induced by Mtb infection and providing important cues for further functional studies. Furthermore, gene ontology (GO) and Kyoto Encyclopedia of Genes and Genomes (KEGG) biological pathway analysis of the differentially expressed mRNAs showed the potential functions and pathways related to the pathogenesis of Mtb infection. Finally, two lncRNAs, MIR3945HG V1 and MIR3945HG V2, were identified as novel candidate diagnostic markers for tuberculosis. Our results provide novel insight into the mechanisms of the pivotal Mtb-macrophage interactions, and reveal potential targets for diagnostics and the treatment of tuberculosis.

*Mycobacterium tuberculosis* (Mtb), the causative agent of tuberculosis, caused approximately 1.5 million deaths in 2014 and surpassed human immunodeficiency virus (HIV) as the top infectious cause of human mortality worldwide^1^. The control of tuberculosis is extremely difficult due to the rapid spread of drug-resistant TB and the low efficacy of the current Bacilli Calmette-Guerin (BCG) vaccine^2^. Therefore, a better understanding of the pathogenesis and host defence mechanisms of tuberculosis would help provide novel and more efficient diagnostic and therapeutic strategies for tuberculosis.

Macrophages play a key role in the host immune system by phagocytosing and eliminating pathogens, as well as initiating protective acquired immune responses via antigen presentation to T cells^3^. Paradoxically, macrophages may be the primary long term residence for Mtb in the host, because Mtb is able to evade the major antimicrobial mechanisms of macrophages by a variety of bacterial immune subversion mechanisms, allowing the bacteria to survive in cell via the utilization of various intracellular resources^3^,^4^,^5^,^6^. Interactions between macrophages and Mtb alters the cellular gene expression profiles, and investigation of these changes in gene expression may help to further our understanding of the regulation of anti-mycobacterial immunity in macrophages. More importantly, novel biomarkers for diagnosis and therapeutic targets for tuberculosis therapy may be revealed. Different Mtb strains induce diverse host responses and ultimate fates^7^. For example, Macrophages infected with avirulent Mtb are inclined to apoptosis, which facilitates clearance of the infection and antigen presentation^8^. In contrast, infection with virulent Mtb leads to macrophage necrosis and the spreading of pathogens into the surrounding tissues^9^. Therefore, comparing the expression profiles of macrophages infected with avirulent H37Ra and virulent H37Rv Mtb strains will help to obtain a greater understanding of the molecular mechanisms involved in Mtb-macrophage interaction.

Among the altered gene expression profiles induced by Mtb infection, both mRNA and long noncoding RNAs (lncRNAs) are important components. Functions of mRNAs could be revealed through gene ontology (GO) and Kyoto Encyclopedia of Genes and Genomes (KEGG) analysis, while lncRNAs represent a new field in tuberculosis immunology. LncRNAs are arbitrarily defined as transcripts longer than 200 nt in length without protein-coding capacity^10^. In recent years, an increasing number of studies have suggested that lncRNAs have a central role in fundamental biological processes, such as genomic imprinting, chromosome modification, immune response, tumorigenesis, cellular development and metabolism, etc^11^,^12^,^13^,^14^,^15^. Emerging evidence also demonstrates the potential of lncRNAs as novel biomarkers and therapeutic targets for many diseases^16^,^17^. However, the function of lncRNAs in regulating the immune response of macrophages to stimuli is just beginning to be understood. For example, lincRNA-Cox2 regulates both the activation and repression of distinct classes of immune response genes (IRGs) in myeloid cells upon TLR ligand stimuli^18^. LncRNA-THRIL is able to modulate TNF-α and other inflammatory gene expression in THP-1 macrophages^19^. Meanwhile, the uncontrolled inflammatory responses in macrophages exposed to microbial ligands can be restrained by LincRNA-EPS via regulation of IRGs expression^20^. Nevertheless, the alteration in lncRNA expression induced by Mtb and the roles of these transcripts in modulating the macrophage response to infection remain unclear.

In this study, we analysed the gene expression profiles of human macrophages at 72 h post infection with H37Ra or H37Rv using microarrays. Our results showed that compared with uninfected cells, and between H37Ra and H37Rv infection, there were great changes in the expression of lncRNAs and mRNAs in macrophages. Further, GO analysis and KEGG pathway analysis performed on the differentially expressed mRNAs suggested that different pathways and functions are involved in the macrophage response to avirulent and virulent Mtb strain infection. More importantly, two lncRNAs, lncRNA-MIR3945HG V1, and MIR3945HG V1, have been demonstrated to be potential biomarkers for tuberculosis diagnosis. Collectively, our study demonstrated substantial alteration in lncRNA and mRNA expression induced by Mtb infection, suggesting that play an important role in the regulation of the immune response to Mtb and may have potential as novel biomarkers for the diagnosis of tuberculosis.

## Results

### Differential expression of lncRNAs in human macrophages between the uninfected, H37Ra and H37Rv groups

**Figure 1:**
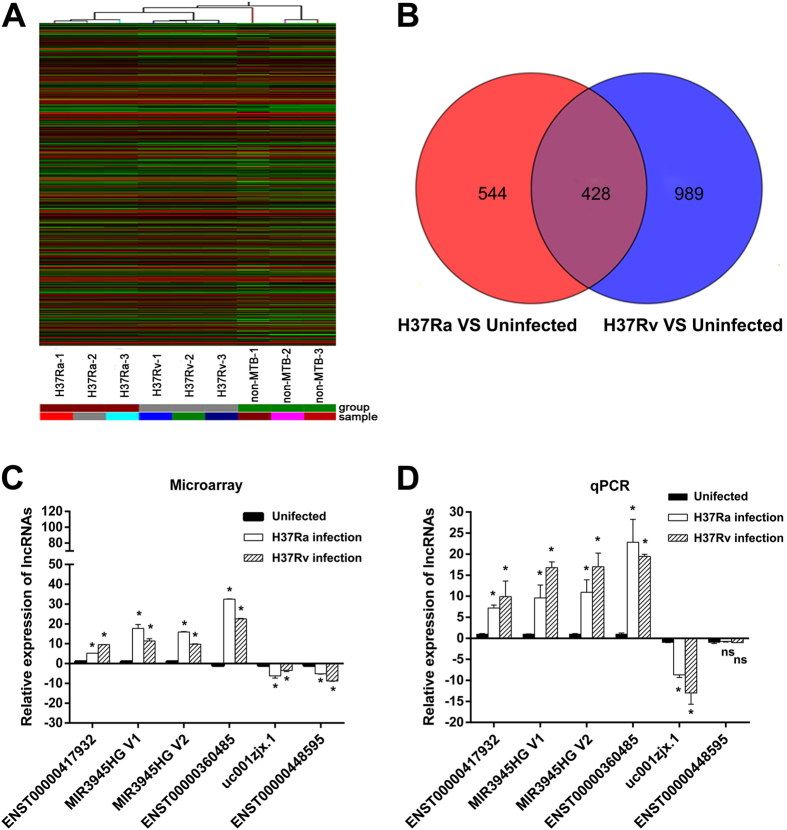
Differential expression of lncRNAs in human macrophages between the uninfected group, the H37Ra group and the H37Rv group. (**A**) The hierarchical clustering of differentially expressed lncRNAs. “H37Ra-1, -2, -3”, “H37Rv-1, -2, -3” and “non-MTB” were samples from three healthy donors’ macrophages infected with H37Ra or H37Rv, or without Mtb infection. In the cluster heat map, red indicates high relative expression, and green indicates low relative expression. (**B**) Venn diagrams indicated the numbers of overlapping and nonoverlapping differentially expressed lncRNAs in macrophages after H37Ra or H37Rv infection compared with macrophages without infection, respectively. (**C**) and (**D**) Differential expression results of sampling lncRNAs from Microarray analysis (**C**) and validated with qPCR (**D**). *P < 0.05, ns indicates non-significant difference.

To evaluate the lncRNA expression profile of human macrophages in response to virulent or avirulent *Mycobacterium tuberculosis* infection, microarray analyses were performed on human macrophages infected with H37Rv or H37Ra. To identify differentially expressed lncRNAs, genes with more than two-fold expression changes (*P* < 0.05) were selected. Hierarchical clustering indicates the differentially expressed lncRNAs (Fig. 1A) among the uninfected group, the H37Ra group and the H37Rv group.

Compared with the uninfected group, there were 972 lncRNAs (487 upregulated, 485 downregulated) that were differentially expressed in the H37Ra group; whereas 1417 lncRNAs (492 upregulated, 925 downregulated) were differentially expressed in the H37Rv group (fold change (FC) ≥ 2 and *P* < 0.05). Compare with the uninfected group, the top 30 deregulated lncRNAs from the H37Ra and H37Rv group are listed in Table 1. Moreover, compare with the uninfected group, 428 lncRNAs were deregulated in both the H37Ra group and H37Rv group. However, 544 lncRNAs and 989 lncRNAs were specifically deregulated in the H37Ra and H37Rv groups, respectively (Fig. 1B).

To validate our microarray data, 6 lncRNAs were randomly selected for detection by quantitative real-time PCR (qPCR). To avoid the effects of individual differences, we recruited another three health volunteers to obtain samples different from the samples used for the microarray experiments. In this validation, 5 of the 6 lncRNAs had the same pattern of expression upon Mtb infection as that determined by microarray analysis (Fig. 1C,D). The discrepancy between the expression pattern of lncRNAs identified by microarray and by qPCR could be explained by potentially inaccurate mapping of lncRNA sequences assembled from available cDNA libraries^19^, thereby increasing false-positive signals by some of the microarray probes.

### Differential expression of mRNAs in human macrophages between the uninfected, H37Ra and H37Rv groups

**Figure 2:**
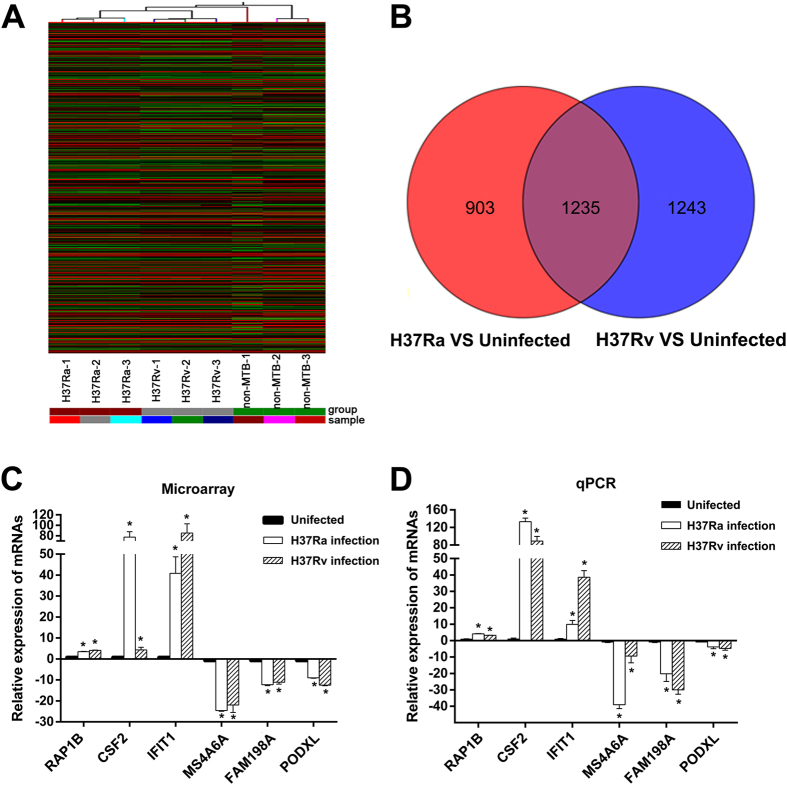
Differential expression of mRNAs in human macrophages between the uninfected group, the H37Ra group and the H37Rv group. (**A**) The hierarchical clustering of differentially expressed mRNAs. (**B**) Venn diagrams indicated the numbers of overlapping and nonoverlapping differentially expressed mRNAs in macrophages after H37Ra or H37Rv infection compared with macrophages without infection, respectively. (**C**) and (**D**) Differential expression results of sampling mRNAs from Microarray analysis (**C**) and validated with qPCR (**D**). *P < 0.05.

Microarray analyses were also performed to examine the expression profile of mRNA in human macrophages infected with H37Rv or H37Ra. Hierarchical clustering indicates the differentially expressed mRNAs (Fig. 2A) among the uninfected group, the H37Ra group and the H37Rv group. In the microarray analysis, 2138 mRNAs and 2478 mRNAs were differentially expressed in the H37Ra and H37Rv groups (FC ≥ 2 and *P* < 0.05), respectively, compared with Uninfected group. The top 30 deregulated mRNAs in the H37Ra and H37Rv groups are listed in Table 2. In the H37Ra group, 1605 mRNAs were upregulated, and 533 mRNAs were downregulated, whereas 1683 mRNAs were upregulated and 795 mRNAs were downregulated in the H37Rv group. It is worth noting that compared with the uninfected group, 1235 mRNAs were differentially expressed in both the H37Ra group and the H37Rv group. Moreover, 903 mRNAs and 1243 mRNAs were specifically deregulated in the H37Ra group and H37Rv group, respectively (Fig. 2B).

To validate our microarray data, 6 lncRNAs were randomly selected for detection by qPCR. The qPCR results showed that compared with the uninfected, group, all of the mRNAs had the same expression trend in both the H37Ra group and H37Rv group as the results obtained by microarray analysis (Fig. 2C,D).

### GO analysis

**Figure 3:**
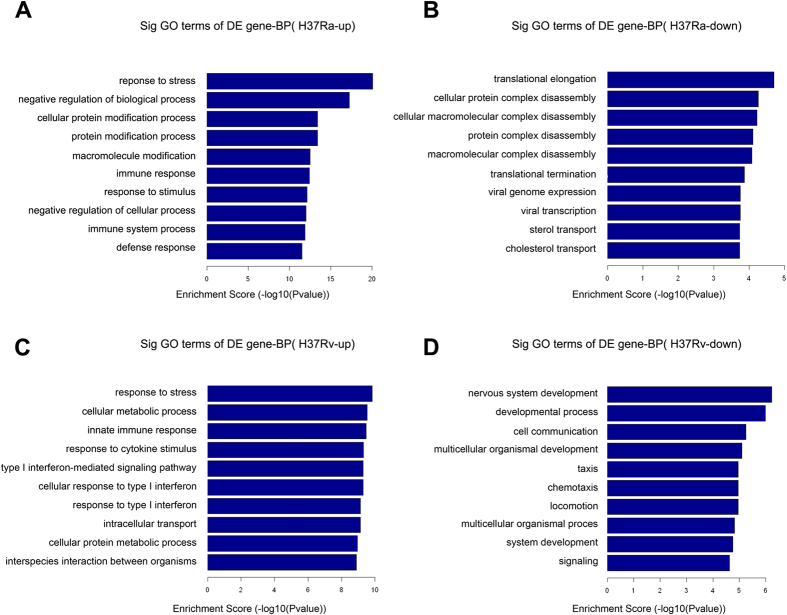
GO analyses of differentially expressed mRNAs according to biological process (BP). The most significant GO terms for upregulated genes (**A**) and downregulated genes (**B**) in macrophages after H37Ra infection. The most significant GO terms for upregulated genes (**C**) and downregulated genes(**D**) in macrophages after H37Rv infection. The GO terms were filtered in accordance with *P* < 0.05 and FDR <0.05. The top 10 significantly enriched GO terms are shown.

GO analysis was performed to gain insight into the potential functions of the Mtb-induced host genes in the macrophages. Differentially expressed mRNAs from the microarray analysis were classified into different functional categories based on the biological processes (BP) of the gene ontology classification. The number of significantly enriched GO terms that indicated upregulated mRNAs in the H37Ra and H37Rv groups were 825 and 580, respectively. Remarkably, 411 of these GO terms were shared between the H37Ra group and the H37Rv group. In contrast, 230 and 339 GO terms of downregulated mRNAs were enriched in the H37Ra group and the H37Rv group, respectively, but only 55 of these GO terms were enriched in both group. The GO analysis showed that compared with the uninfected group, the upregulated mRNAs in the H37Ra group were mainly involved in “response to stress”, “negative regulation of biological process”, “cellular protein modification process”, “protein modification process”, and “macromolecule modification”; the downregulated mRNAs were mainly involved in “translational elongation”, “cellular protein complex disassembly”, “cellular macromolecular complex disassembly”, “protein complex disassembly”, and “macromolecular complex disassembly”, etc (Fig. 3A,B). Conversely, compared with the uninfected group, the upregulated mRNAs in the H37Rv group were significantly enriched in “response to stress”, “cellular metabolic process”, “innate immune response”, “response to cytokine stimulus”, “type I interferon-mediated signalling pathway”; the downregulated mRNAs were primarily involved in “nervous system development”, “developmental process”, “cell communication”, “multicellular organismal development”, “taxis”, etc (Fig. 3C,D).

### KEGG pathway analysis

**Figure 4:**
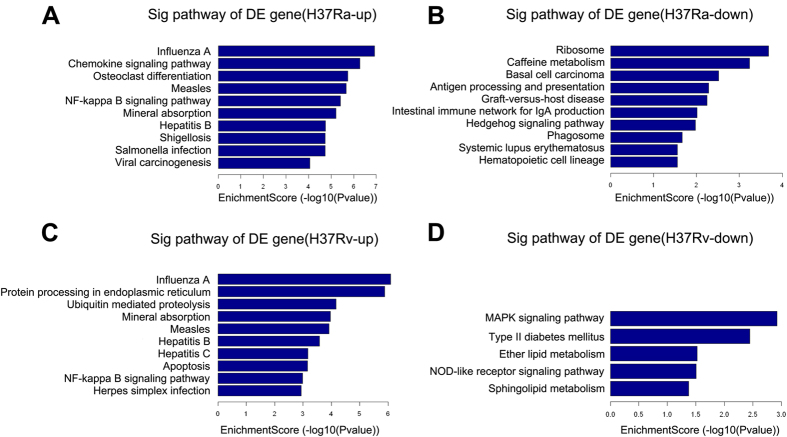
KEGG pathways analysis of differentially expressed mRNAs. The most significant pathways for upregulated genes (**A**) and downregulated genes (**B**) in macrophages after H37Ra infection. The most significant pathways for upregulated genes (**C**) and downregulated genes (**D**) in macrophages after H37Rv infection.

KEGG Pathway analysis was used to investigate the involved biological pathways of the differentially expressed mRNAs. The numbers of pathway terms that the upregulated mRNAs in the H37Ra and H37Rv groups were involved in were 65 and 45, respectively. Remarkably, 35 of these pathway terms were shared between the two groups. In contrast, 13 and 5 pathway terms of downregulated mRNAs were independently enriched in the H37Ra group and the H37Rv group, respectively. The KEGG pathway analysis showed that compared with the uninfected group, the upregulated mRNAs in the H37Ra group mainly participated in “Influenza A”, “Chemokine signaling pathway”, “Osteoclast differentiation”, “Measles”, and “NF-kappa B signaling pathway”, whereas the downregulated mRNAs were significantly involved in “Ribosome”, “Caffeine metabolism”, “Basal cell carcinoma”, “Antigen processing and presentation”, and “Graft-versus-host disease” (Fig. 4A,B). However, compared with the uninfected group, the upregulated mRNAs in H37Rv group were significantly associated with “Influenza A”, “Protein processing in endoplasmic reticulum”, “Ubiquitin mediated proteolysis”, “Mineral absorption”, “Measles”, whereas downregulated mRNAs were mainly enriched in “MAPK signaling pathway”, “Type II diabetes mellitus”, “Ether lipid metabolism”, “NOD-like receptor signaling pathway”, and “Sphingolipid metabolism” (Fig. 4C,D).

### Identification of lncRNAs as candidate biomarkers for tuberculosis

**Figure 5:**
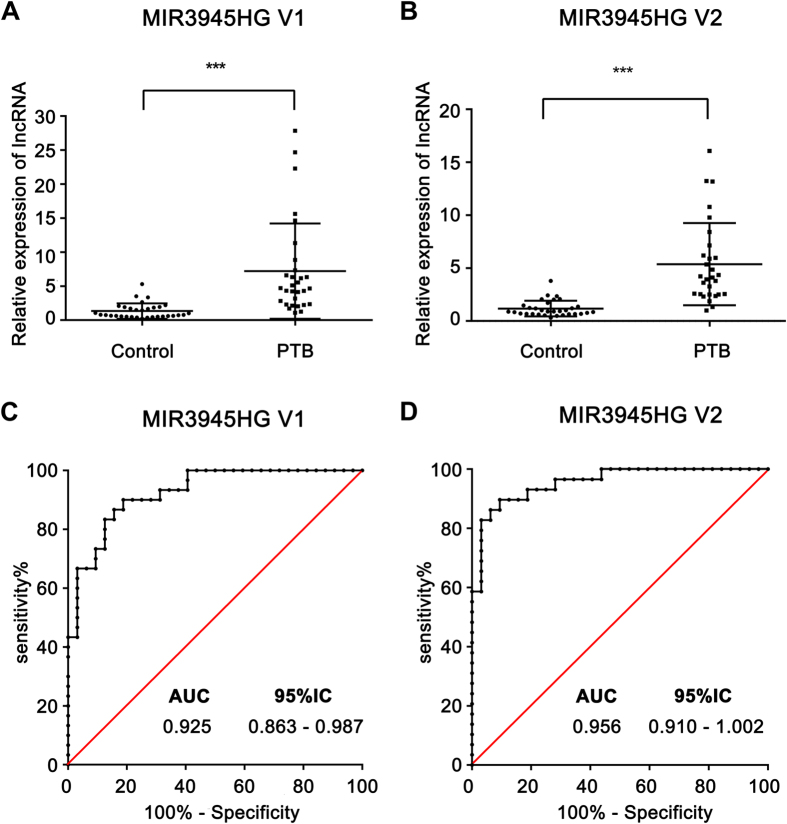
Assessment of the diagnostic value of MIR3945HG V1 and MIR3945HG V2 for tuberculosis. The qPCR results of MIR3945HG V1 (**A**) and MIR3945HG V2 (**B**) in active pulmonary tuberculosis patients compared with healthy control. Receiver operating characteristic (ROC) curve for MIR3945HG V1 (**C**) and MIR3945HG V2 (**D**). AUC: Area under the ROC curve. ***P < 0.001

There is an urgent need for the identification of novel and more efficient diagnostic methods to aid in tuberculosis control. In recent years, many studies have demonstrated that lncRNAs can serve as novel diagnostic markers for a variety of diseases. Thus, we sought to determine whether the differentially expressed lncRNAs identified in our microarray analysis could potentially serve as diagnostic biomarkers for tuberculosis. The expression level of four lncRNAs, ENST00000360485, ENST00000417932, MIR3945HG V1 and MIR3945HG V2, which were selected based on their significant upregulation in macrophages with Mtb infection *in vitro*, were detected by qPCR in PBMC samples from 31 patients with active pulmonary tuberculosis (PTB patients) and 32 healthy people vaccinated with BCG (Table 3). The qPCR results showed that compare with the healthy controls, the expression of ENST00000360485 was downregulated in the PTB patients (*P* < 0.001) (Supplementary Fig. S1A), while the expression of MIR3945HG V1 (NCBI Reference Sequence: NR_037867; Homo sapiens MIR3945 host gene (MIR3945HG), transcript variant 1) and MIR3945HG V2 (NCBI Reference Sequence: NR_132989.1; Homo sapiens MIR3945 host gene (MIR3945HG), transcript variant 2) were both significantly elevated in the PTB patients (*P* < 0.001) (Fig. 5A,B). However, there was no significant difference in the expression of ENST00000417932 (Supplementary Fig. S2). Furthermore, ROC analysis was performed to evaluate the predictive power of ENST00000360485, MIR3945HG V1 and MIR3945HG V2. The sensitivity, specificity, and AUC (area under the ROC curve) of the three candidate biomarkers are shown in Table 4. From Table 4, compared with ENST00000360485 (0.7984; 95% CI: 0.687–0.909), the AUC was significantly larger for MIR3945HG V1 (0.925; 95% CI:0.863–0.987) and MIR3945HG V2 (0.956; 95% CI: 0.910–1.002) in patients with active pulmonary tuberculosis versus healthy controls. It is well known that if the AUC is over 0.9 that indicated diagnostic tests are excellent. Thus, MIR3945HG V1 and MIR3945HG V2 may be effective diagnostic biomarkers for tuberculosis.

## Discussion

The interaction between macrophages and Mtb remains to be clearly determined. In recent years, lncRNA and mRNA expression profiles have been used widely to uncover the underlying molecular mechanisms contributing to pathogenesis of many human diseases, such as cancers^21^, neurological disorders^22^, infectious diseases^23^. In this study, we first investigated the expression patterns of lncRNAs and mRNAs in human macrophages after 72 h infection with H37Ra or H37Rv. Compared with the uninfected group, the expression profiles of lncRNA and mRNA were profoundly altered in the infected groups. Furthermore, GO and KEGG pathway analysis of the differentially expressed mRNAs revealed some of the potential functions and pathways related to the pathogenesis of Mtb infection. Finally, two lncRNAs, MIR3945HG V1 and MIR3945HG V2, were identified as the novel candidate diagnostic markers for tuberculosis. Collectively, our results provide novel insight into the mechanisms of the pivotal Mtb-macrophage interactions during the late stage of infection.

In our study, we found profoundly different expression patterns of lncRNA and mRNA between macrophages infected with H37Rv or H37Ra compared with uninfected cells, and many more genes were differentially expressed in H37Rv-infected macrophages than in H37Ra-infected cells (Figs 1 and 2). This observation was consistent with the results from the similar studies performed using human alveolar macrophages^24^. Moreover, it is worth noting that in contrast to the similar number of lncRNAs or mRNAs up-regulated by H37Rv and H37Ra, the numbers of lncRNAs and mRNAs down-regulated by H37Rv were far greater than those downregulated by H37Ra. These results indicated that virulent strains may promote their intracellular survival primarily via repressing the expression of particular genes in macrophages.

GO analysis and KEGG pathway analysis were performed to gain insight into the potential functions of the differentially expressed mRNAs and to improve our understanding of the mechanisms of the Mtb–macrophage interaction during the late infection stage. In our results, most of the enriched upregulated GO terms and pathway terms were shared together between the H37Ra and the H37Rv groups. In contrast, the significantly enriched downregulated GO terms and pathway terms in the H37Ra group and the H37Rv group are quite different from each other. These data suggested that virulent Mtb strains may evade macrophage defences via the repression of particular pathways. For example, in our data, the MAPK signalling pathway and lipid metabolism pathway (“ether lipid metabolism” and “sphingolipid metabolism”) were inhibited in macrophages infected with H37Rv (Fig. 4D). The MAPK signalling pathway is essential for regulating the expression of Mtb-induced immunoregulatory molecules, such as TNF-α, IL-1β, and MCP-1^25^,^26^, while perturbations in the host lipid metabolism of macrophages have been reported in Mtb infection as one of the key strategies for Mtb persistence^27^. Intriguingly, the NF-kappa B pathway, which regulates innate immunity via modulating the expression of immunoregulatory molecules^28^, was enriched in upregulated mRNAs in the H37Rv group. A recent study demonstrated that inhibition of the NF-kappa B pathway was critical for persistence of Mtb^29^. Therefore, the outcome of macrophages infected with Mtb depends on the balance between the host defence of macrophages and bacterial immune subversion mechanisms.

Although the study of lncRNAs is a hot topic, the role of lncRNAs in the pathogenesis of tuberculosis is just beginning to be investigated. Recently, Zhengjun Y. *et al*. employed microarray analysis to detect expression profile of lncRNA and mRNA in CD4^+^ T cells from individuals with latent TB infection (LTBI), active TB and healthy controls^30^. These authors’ revealed that lncRNAs were differentially expressed in CD4^+^ T cells in response to Mtb infection and suggested that these transcripts may play an important role in regulating the host immune response to Mtb infection. Wang, Y. *et al*. reported that lncRNA-CD244 acted as an epigenetic regulator of IFN-γ and TNF-α production in CD8^+^ T cells and that it impacts CD8^+^ T cell immunity against active Mtb infection^31^. LncRNA-MEG3, which is shown to be a tumour suppressor gene, was downregulated in macrophages with BCG infection, leading to the induction of autophagy and enhanced eradication of intracellular pathogens^32^. In this study, we revealed that many lncRNAs were differentially expressed in macrophages with H37Ra or H37Rv infection, which indicated that lncRNAs might play an important role in the macrophages response to Mtb infection. One recent study implied that the lncRNA MT1JP played a potential role in promoting apoptosis to restrain the growth of tumour cells^33^. In our microarray data, the expression of MT1JP in macrophages with H37Ra infection was significantly higher than that with H37Rv infection (Table 1). MT1JP might contribute to apoptosis induced by H37Ra, but this contribution requires further research to be conclusive. Most of the lncRNAs in our data have not been investigated. Fully exploring the role of these lncRNAs induced by Mtb would provide new insights into tuberculosis pathogenesis.

Effective diagnosis of Mtb infection is critical for the treatment of tuberculosis and the control of tuberculosis transmission. However, developing an accurate and rapid diagnosis of active tuberculosis infection is difficult^34^. In recent years, increasing evidences have suggested that lncRNAs can serve as novel diagnostic markers for many diseases. For example, a previous report showed that a circulating lncRNA OTTHUMT00000387022 from monocytes can be used as a novel biomarker for coronary artery disease^35^. Circulating lncRNA-HULC may be a candidate serum tumour marker for the early diagnosis of gastric cancer and for monitoring its progression and prognosis^36^. LncRNA PCA3 in patient urine samples was validated as a more specific diagnostic marker for prostate cancer than the widely used prostate-specific antigen^37^. However, there are no reports regarding the use of lncRNAs as biomarkers for tuberculosis. In our microarray data, there were many differentially expressed lncRNAs induced by Mtb infection, which indicated that lncRNAs have the potential to be novel biomarkers for tuberculosis. In this study, we found 3 lncRNAs, ENST00000360485, MIR3945HG V1 and MIR3945HG V2, that in comparison with healthy donors vaccinated with BCG were significantly aberrantly expressed in PBMC samples from patients with active pulmonary tuberculosis (Supplementary Fig. S1A, Fig. 5A,B). ROC analysis showed that AUC of MIR3945HG V1 and MIR3945HG V2 were over 0.9, while that of ENST00000360485 was lower than 0.8 (Table 4), which indicates that MIR3945HG V1 and MIR3945HG V2 may function as more promising candidate biomarkers for tuberculosis diagnosis. However, a larger sample size is needed to confirm our results. The functions of MIR3945HG V1 and MIR3945HG V2 are still unknown. A recent study showed that the role of lncRNA MIR31HG in biological processes is independent of miR-31, which maps to the intronic region of MIR31HG^38^. Likewise, the function of MIR3945HG may be independent of miR-3945, which maps to both MIR3945HG V1 and MIR3945HG V2, but further investigation is required.

In summary, our study revealed that a great many lncRNAs and mRNAs were consistently induced during macrophage infection with H37Ra or H37Rv. This study also suggested that lncRNAs might be crucial for regulating the antimicrobial mechanisms of macrophages. More importantly, we demonstrated that two lncRNAs, MIR3945HG V1 and MIR3945HG V2, have the potential to be novel diagnostic biomarkers for tuberculosis. However, the underlying molecular mechanisms of the pivotal Mtb-macrophage interactions still require further study. As the relationship between lncRNAs and Mtb infection is just beginning to be investigated, additional characterization of differentially expressed lncRNAs in macrophages would expand our understanding of tuberculosis pathogenesis.

## Methods

### Ethical statement

The study was approved by the Ethics Committee of Southern Medical University and conducted in accordance with the Declaration of Helsinki. Written informed consent was obtained from all participants before the commencement of the study.

### Sample collection

The peripheral blood samples (3 mL) used to validate candidate biomarkers were collected from 31 patients with active pulmonary tuberculosis and 32 healthy donors. All patients with active pulmonary tuberculosis were diagnosed based on typical clinical symptoms, chest radiography, sputum staining for acid-fast bacilli (AFB), and positive culture and PCR for Mtb in the Guangzhou Chest Hospital in Guangzhou, China. All patients had primary tuberculosis and were not undergoing anti-TB treatment at the time of analysis. Healthy controls were not in contact with TB patients and were free of clinical signs of tuberculosis or latent tuberculosis infection. Individuals with cancer, allergic diseases, immune-compromised conditions, diabetes or other infectious diseases such as HBV, HCV and HIV infection were excluded. After sample collection, peripheral blood mononuclear cells (PBMCs) were isolated by density gradient centrifugation using Hypaque-Ficoll (GE Healthcare Bio-sciences AB, Uppsala, Sweden) according to the manufacturer’s protocol. Then, the PBMC samples were lysed with TRIzol^®^ Reagent (Invitrogen, Carlsbad, CA, USA) and stored at −80 °C.

### Cell culture and infection

Peripheral blood samples (200 mL) were collected from 6 healthy donors. Then, PBMCs were isolated by the above methods. Monocytes were purified from PBMC by positive selection with CD14^+^ magnetic bead (miniMACS, Miltenyi Biotec, Gladbach, Germany) and were differentiated into monocyte derived macrophages (MDMs) by treating with macrophage colony-stimulating factor (GM-CSF) as previously described^39^. Briefly, CD14^+^ monocytes were culture in RPMI-1640 (Corning, NY, USA) containing 10% fetal bovine serum (FBS) and 1000 U/mL of GM-CSF for 7 days, and the medium was half-changed every 48 hours. Furthermore, the macrophages were identified by morphologic observation and flow cytometric analysis followed by anti-CD68 staining^40^.

Macrophages were incubated with *M. tuberculosis* H37Rv (27294) or H37Ra (25177) at an MOI of 10 (10 bacteria: 1 cell). After 4 h of incubation at 37 °C, extracellular bacteria were removed by washing the culture with phosphate-buffered saline (PBS). Cells were cultured in RPMI-1640 containing 10% FBS without antibiotics at 37 °C in 5% CO_2._ After 72 h, uninfected macrophages and MTB infected macrophages were lysed with TRIzol^®^ Reagent. *M. tuberculosis* strains H37Rv (27294) and H37Ra (25177) strains were provided by Beijing Research Institute for Tuberculosis Control. Before infecting macrophages, *M. tuberculosis* was dispersed to single cells.

RNA samples from 3/6 subjects were prepared for microarray analysis, and the others were used to validate the microarray data with qPCR.

### RNA extraction and quality control

Total RNA was extracted using TRIzol^®^ Reagent according to the manufacturer’s protocol. Total RNA from each sample was quantified using a NanoDrop ND-1000, and RNA integrity was assessed by standard denaturing agarose gel electrophoresis.

### Microarray analysis of lncRNA and mRNA expression

An Arraystar Human LncRNA Microarray V3.0 (Array-Star, Inc., Rockville, MD, USA) was employed in this study. This array contains 30,586 LncRNA probes and 26,109 coding transcript probes, and it was constructed using the most authoritative public transcriptome databases (e.g., Refseq, UCSC knowngenes, Gencode), as well as highly-respected publications (more detailed information about the microarray is available at www.arraystar.com/microarray/service_main.asp?id=198). The Agilent Array platform was used for the microarray analysis. The sample preparation, microarray hybridization, slide washing and scanning were performed according to the standard Arraystar protocols with minor modifications. Agilent Feature Extraction software (version 11.0.1.1, Agilent Technologies) was employed to analyse the acquired array images. Quantile normalization and further data processing were performed using the GeneSpring GX v11.5 software package (Agilent Technologies). Volcano Plot filtering was used to distinguish the significantly differentially expressed LncRNAs and mRNAs between the two groups. Differentially expressed LncRNAs and mRNAs were identified through Fold Change filtering. The microarray work was completed by KangChen Bio-tech, Shanghai, People’s Republic of China.

### Quantitative real-time PCR (qPCR)

Following RNA extraction, 1 μg of RNA samples were reverse transcribed into cDNA using TransScript One-Step gDNA Removal and cDNA Synthesis SuperMix (TransGen Biotech, Beijing, China) with oligo-dT primer according to the manufacturer’s protocols. Real-time PCR was performed using TransStart Top Green qPCR SuperMix (TransGen Biotech, Beijing, China) on a Mastercycler ep realplex4 (Eppendorf, Hamburg, Germany). The PCR conditions included an initial step at 95 °C for 30 s, followed by 40 cycles of amplification and quantification (95 °C for 15 s, 60 °C for 15 s, and 68 °C for 20 s). GAPDH was used as an internal control. Relative gene expression levels were calculated using the 2^−ΔΔCt^ method^41^. The sequences of the primers used are listed in Supplementary Table S1. Melt curve analysis was performed to verify the specificity of primers.

### GO and pathway analysis

Gene ontology (GO) analysis (www.geneontology.org) was used to investigate biological functions based on differentially expressed coding genes^42^. This analysis classifies functions according to the three following aspects: biological process, cellular component and molecular function. Fisher’s exact test was applied to classify the GO category. The p-value denotes the significance of GO term enrichment in the deregulated expressed genes. The lower the p-value, the more significant the GO term (*P* value *<* 0.05 is recommended).

Pathway analysis was used to investigate the differentially expressed coding genes according to the Kyoto Encyclopedia of Genes and Genomes (KEGG), Biocarta and Reactome (http://www.genome.jp/kegg/)^43^. The p-value (EASE-score, Fisher-*P*value or Hypergeometric-*P* value) indicates the significance of the pathway correlated with the conditions. *P* < 0.05 was considered statistically significant.

### Statistical analysis

All results were presented as the mean ± standard error (SE) of three independent experiments. A one-way ANOVA test or unpaired t-test was used for statistical analysis. Receiver operating characteristic (ROC) analysis was used to evaluate the power of each candidate biomarker. All statistical tests were performed with GraphPad Prism 6.0 (GraphPad Software Inc., La Jolla, CA, USA). All statistical tests were two tailed, and *P* < 0.05 was considered significant.

## Additional Information

**How to cite this article**: Yang, X. *et al*. Microarray analysis of long noncoding RNA and mRNA expression profiles in human macrophages infected with *Mycobacterium tuberculosis. Sci. Rep.* **6**, 38963; doi: 10.1038/srep38963 (2016).

**Publisher's note:** Springer Nature remains neutral with regard to jurisdictional claims in published maps and institutional affiliations.

## Supplementary Material

Supplementary Information

## Figures and Tables

**Table 1:** The top 30 differentially expressed lncRNAs between the Mtb infected group and the uninfected group.

Rank	H73Ra group vs uninfected group			H73Rv group vs uninfected group		
	Seqname	GeneSymbol	Fold change	Seqname	GeneSymbol	Fold change
1	uc001hyk.1	BC133032	169.28	uc001hyk.1	BC133032	70.83
2	ENST00000514265	RP11-184M15.1	−38.31	ENST00000540392	RP11-283G6.4	21.73
3	uc021ybu.1	HM358988	29.60	ENST00000541940	RP11-283G6.5	19.63
4	NR_036682	BCL7B	21.12	NR_026913	LOC100133669	17.24
5	ENST00000444023	MT1JP	20.58	ENST00000551898	RP11-493L12.2	−16.67
6	NR_024280	UNQ6494	20.10	ENST00000536112	RP11-81H14.2	15.51
7	ENST00000551898	RP11-493L12.2	−19.25	ENST00000440492	RP11-288L9.1	15.25
8	ENST00000360485	RP11-81H14.2	18.89	ENST00000567533	RP11-90P13.1	14.45
9	ENST00000503066	RP11-291L15.2	17.95	ENST00000541707	RP11-81H14.2	13.94
10	ENST00000536112	RP11-81H14.2	17.72	NR_046398	XAF1	13.35
11	ENST00000540392	RP11-283G6.4	17.58	ENST00000360485	RP11-81H14.2	13.03
12	ENST00000541940	RP11-283G6.5	16.68	ENST00000448595	AC109826.2	−12.25
13	TCONS_00029017	XLOC_013940	16.00	ENST00000540625	RP11-283G6.5	11.90
14	ENST00000541707	RP11-81H14.2	15.24	uc002iby.2	LOC388387	−10.80
15	uc022blc.1	DQ580140	14.92	TCONS_00004488	XLOC_002383	-10.62
16	NR_026913	LOC100133669	14.71	ENST00000429482	RP11-85O21.5	−9.941
17	ENST00000429482	RP11-85O21.5	−13.80	TCONS_00025730	XLOC_012551	9.70
18	ENST00000440492	RP11-288L9.1	12.52	ENST00000417932	RP11-10J5.1	9.49
19	ENST00000497961	RP11-242C19.2	12.17	ENST00000502400	RP11-584P21.2	−9.30
20	uc002iby.2	LOC388387	−12.13	uc021ybu.1	HM358988	9.17
21	NR_037867 (NR_037867.1)	LOC731424 (MIR3945HG V1)	11.81	NR_024280	UNQ6494	8.82
22	uc003iwy.1 (NR_132989.1)	BC016366 (MIR3945HG V2)	11.81	ENST00000511053	KHDC1P1	−8.69
23	ENST00000569685	CTA-331P3.1	−10.99	uc002vja.1	AX748340	8.52
24	ENST00000486551	MT1DP	10.66	ENST00000456985	RP11-525A16.4	−7.97
25	ENST00000567054	MT1CP	10.15	NR_037867 (NR_037867.1)	LOC731424 (MIR3945HG V1)	7.55
26	ENST00000567533	RP11-90P13.1	9.82	ENST00000499202	RP5-940J5.8	−7.43
27	ENST00000420563	AC053503.4	−9.35	ENST00000421965	AC006159.4	−7.35
28	uc002vja.1	AX748340	9.24	uc003iwy.1 (NR_132989.1)	BC016366 (MIR3945HG V2)	7.18
29	NR_046398	XAF1	9.07	TCONS_00020439	XLOC_009769	7.04
30	ENST00000540625	RP11-283G6.5	9.04	TCONS_00025843	XLOC_012114	−6.99

Positive value and negative of fold change indicated upregulation and downregulation, respectively. p-value calculated from *t*-test and statistical significance was defined as *P* < 0.05. lncRNAs hybridized with nonspecific probe were excluded.

**Table 2:** The top 30 differentially expressed mRNA between Mtb infected group and the uninfected group.

Rank	H73Ra group vs Uninfected group			H73Rv group vs Uninfected group		
	Seqname	GeneSymbol	Fold change	Seqname	GeneSymbol	Fold change
1	NM_005950	MT1G	172.20	NM_001548	IFIT1	84.17
2	NM_001130046	CCL20	166.83	NM_000129	F13A1	−80.70
3	NM_001185156	IL24	110.24	NM_001565	CXCL10	71.48
4	NM_002090	CXCL3	91.36	NM_005950	MT1G	67.73
5	NM_000758	CSF2	76.92	NM_170780	CD200R1	−45.18
6	NM_001135652	EIF2AK2	75.55	NM_207315	CMPK2	40.76
7	NM_000129	F13A1	−63.81	NM_001135652	EIF2AK2	36.89
8	NM_001135651	EIF2AK2	62.55	NM_001130046	CCL20	35.28
9	NM_170780	CD200R1	−61.40	NM_022873	IFI6	31.77
10	NM_001565	CXCL10	52.18	NM_001135651	EIF2AK2	29.63
11	NM_022873	IFI6	47.55	NM_152851	MS4A6A	−27.95
12	NM_000576	IL1B	47.50	NM_032471	PKIB	−27.41
13	NM_002981	CCL1	46.33	NM_016817	OAS2	26.07
14	NM_001143818	SERPINB2	46.19	NM_152703	SAMD9L	24.72
15	NM_207315	CMPK2	45.49	NM_080657	RSAD2	23.99
16	NM_001548	IFIT1	40.34	NM_002535	OAS2	23.46
17	NM_000265	NCF1	38.13	NM_001006600	ERBB2IP	22.04
18	NM_002038	IFI6	35.41	NM_152852	MS4A6A	−21.34
19	NM_152851	MS4A6A	−34.06	ENST00000307407	IL8	21.28
20	NM_176870	MT1M	33.73	NM_001002264	EPSTI1	20.53
21	NM_032965	CCL15	33.37	NM_002906	RDX	20.43
22	NM_002164	IDO1	33.18	NM_002038	IFI6	19.28
23	NM_004099	STOM	32.68	NM_004099	STOM	18.42
24	NM_002089	CXCL2	32.29	NM_014358	CLEC4E	18.35
25	NM_032471	PKIB	−31.26	NM_182742	TXNRD1	15.88
26	NM_002906	RDX	30.19	NM_003522	HIST1H2BF	−15.62
27	ENST00000307407	IL8	30.06	NM_000265	NCF1	14.83
28	NM_152852	MS4A6A	−29.81	NM_017414	USP18	14.59
29	NM_005306	FFAR2	28.80	NM_002758	MAP2K6	−14.01
30	NM_130782	RGS18	−25.60	NM_005101	ISG15	13.68

Positive value and negative of fold change indicated upregulation and downregulation, respectively. *P*-value calculated from t-test and statistical significance was defined as *P* < 0.05.

**Table 3:** The demographic characteristics of the study populations.

Study cohort	n	Median age in years (range)	Gender (M/F)	TST test	IGRA
HC	32	23(19–32)	17/15	0/32	0/32
ATB	31	26(19–35)	13/18	31/31	31/31

TST, Tuber-Culin Skin Test; IGRA, Interferon-Gamma release assays; HC: healthy control; ATB: active pulmonary tuberculosis.

**Table 4:** Performance of candidate biomarkers for detection of active pulmonary tuberculosis.

Candidate biomarkers	AUC (95% CI)	Sensitivity (%) (95% CI)	Specificity (%) (95% CI)	*p* value
MIR3945HG V1	0.925 (0.863–0.987)	90 (73.47–97.89)	81.25 (63.56–92.79)	<0.0001
MIR3945HG V2	0.956 (0.910–1.002)	89.66% (72.65–97.81)	90.63 (74.98–98.02)	<0.0001
ENST00000360485	0.7984 (0.687–0.909)	83.87 (66.27–94.55)	71.88 (53.25 –86.25)	<0.0001
