# Supplementary material for: Microarray analysis of long noncoding RNA and mRNA expression profiles in human macrophages infected with Mycobacterium tuberculosis
Source: Sci Rep. 2016 Dec 14;6:38963. doi: 10.1038/srep38963 (PMC5155227; doi:10.1038/srep38963)
Supplement: Supplementary Information [file srep38963-s1.pdf]

Microarray analysis of long noncoding RNA and mRNA expression profiles in human macrophages infected with *Mycobacterium tuberculosis*

Xiaofan Yang[1], Jiahui Yang[1], Jinli Wang[1], Qian Wen[1], Hui Wang[1], Jianchun He[1], Shengfeng Hu[1], Wenting He[1], Xialin Du[1], Sudong Liu[1], Li Ma[1, 2 *]

1. Institute of Molecular Immunology, School of Biotechnology, Southern Medical University, Guangzhou 510515, China

2. Guangdong Provincial Key Laboratory of Tropical Disease Research, School of Public Health, Southern Medical University, Guangzhou 510515, China

* Correspondence:

Li Ma, M.D., Ph.D.

Institute of Molecular Immunology, School of Biotechnology, Southern Medical University, Guangzhou 510515, China

Tel: 86-20-6164-8322, Fax: 86-20-6164-8322

Email: maryhmz@126.com

Supplementary Table 1. RT-qPCR primers sequences

Name		Sequences	
	qPCR primers	Forward	Reverse
lncRNA	ENST00000417932	CATCAACACAGAAACCTCCGC	CTGACAGAAACGTCAGCCAAG
	NR_037867 （MIR3945HG V1）	GCTGCTATCGCCAAGATCCA	GTGACTTGCGGGAGGAGAAT
	uc003iwy.1 （MIR3945HG V2）	ACATTTGCTGACTGGTCGATG	GCCTTGAATTGGGACATGAGC
	ENST00000360485	CCTCTGGGCAGTGTGATTTG	ATTGCCATACACCGTGGTCAT
	uc001zjx.1	CATCCTGCTACAACTTCCACAA	CTGCTACGAACACTCACATACAT
	ENST00000448595	AACTGGCTGGCTGAATTGGA	TGTGATGATGCTCTTGGTGTTG
mRNA	GAPDH	GAAGACGGGCGGAGAGAAAC	CGACCAAATCCGTTGACTCC
	RAP1B	GTGACAGCGTGAGAGGTACT	AAGCATACACTGTTGTGCATC
	CSF2	GGGAGCATGTGAATGCCATC	GGCTCCTGGAGGTCAAACAT
	IFIT1	AGCCTCCTTGGGTTCGTCTA	AAAGTCAGCAGCCAGTCTCA
	MS4A6A	GGCTCTCTATCAATCGCCAC	CAGAGAGAGAGTTCCACTGC
	FAM198A	TGAGGAGGGACATTACTTTGTCA	AAGCCGGTAAAGCTGCCAT
	PODXL	TCCCAGAATGCAACCCAGAC	GGTGAGTCACTGGATACACCAA

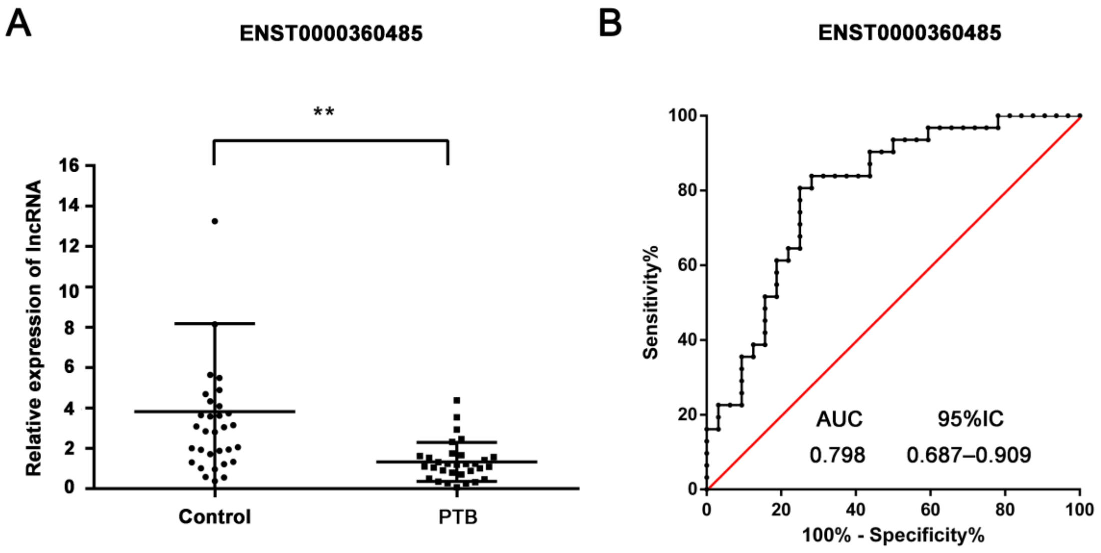

Supplementary Figure S1. Assessment of the diagnostic value of ENST00000360485 for tuberculosis. (A) qPCR result of ENST00000360485 in active pulmonary tuberculosis patients compared with healthy control. (B) Receiver operating characteristic (ROC) curve for ENST00000360485. AUC: Area under the ROC curve. **P< 0.01.

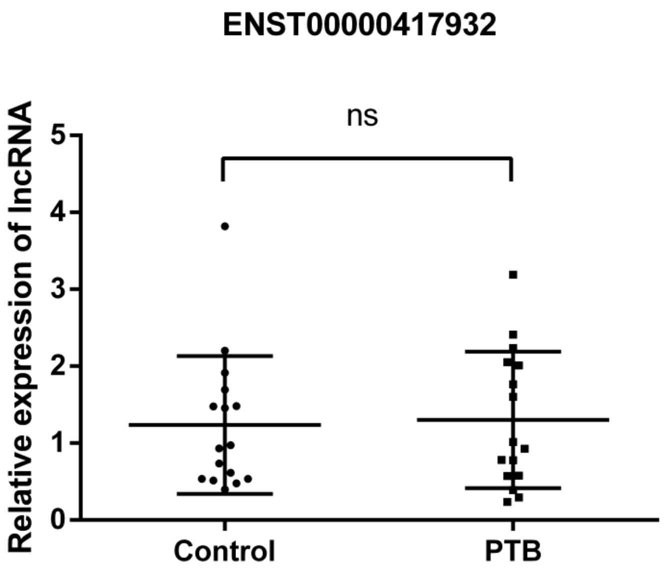

Supplementary Figure S2. Assessment of the diagnostic value of ENST00000417932 for tuberculosis. qRT-PCR result of ENST00000417932. ns indicates non-significant difference.
